# Preventing Undesired Face-Touches With Wearable Devices and Haptic Feedback

**DOI:** 10.1109/ACCESS.2020.3012309

**Published:** 2020-07-27

**Authors:** Nicole D’Aurizio, Tommaso Lisini Baldi, Gianluca Paolocci, Domenico Prattichizzo

**Affiliations:** 1 Department of Information Engineering and MathematicsUniversity of Siena 9313 53100 Siena Italy; 2 Department of Advanced RoboticsIstituto Italiano di Tecnologia (ADVR) 16163 Genova Italy

**Keywords:** Haptic interfaces, magnetic sensors, wearable sensors

## Abstract

The alarming morbidity of COVID-19 has drawn the attention to the social role of hygiene rules, with a particular focus on the importance of limiting face-touch occurrences. To deal with this aspect, we present No Face-Touch, a system able to estimate hand proximity to face and notify the user whenever a face-touch movement is detected. In its complete setup, the system consists of an application running on the smartwatch and a wearable accessory. Its ease of implementation allows this solution to be ready-to-use and large-scale deployable. We developed two gesture detection approaches compatible with sensors embedded in recent smartwatches, i.e. inertial and magnetic sensors. After preliminary tests to tune target gesture parameters, we tested the two approaches and compared their accuracy. The final phase of this project consisted in exploiting the most robust approach in a daily living scenario during a 6-days campaign. Experimental results revealed the effectiveness of the proposed system, demonstrating its impact in reducing the number of face-touches and their duration.

## I. Introduction

The epidemic of severe acute respiratory syndrome coronavirus 2 (SARS-CoV-2) has caused 10 357 662 laboratory-confirmed infections including 508 055 deaths all over the world by 1st July 2020 [1]. Among other factors, the speed of the outbreak has inevitably provoked national and global public health crisis. Not only has coronavirus disease (COVID-19) an alarming morbidity and mortality, but it has also an extended incubation period and a high variability in symptoms manifestation, which result in important implications for surveillance and control activities [2].

Among the policies carried out in response to COVID-19, individual protective behaviour has a great significance on the reduction of the index $R_{0}$, i.e. the average number of infections caused by a primary case in a population consisting only of susceptibles [3]. As a general rule, protective behaviours can be classified into three groups: preventive, avoidant, and management of disease [4]. The first group includes hygiene measures (such as hand washing, cough and sneeze etiquette, and surfaces cleaning), mask wearing and uptake of vaccinations. Observance of these behaviours has effects mainly on the risk of transmission factor. Avoidant behaviours are mostly represented by social distancing, e.g. avoid going to crowded places, maintaining at least 1 m distance between ourself and others, working in compliance with quarantine restrictions. The last category includes following the directions of local health authority when seeking medical attention and staying home and self-isolate even with minor symptoms. In response to a pandemic flu, respecting hygiene measures becomes even more valuable in case virus transmission can occur by self-inoculation, i.e. by transferring contaminated material from hands to other body sites [5], [6]. Although the literature on the mechanisms of self-inoculation of common respiratory infections (e.g., influenza, coronavirus) is limited [7]–[9], contaminated hands have been reported as having potential to disseminate respiratory infections [10], especially if associated to face-touches [11]. As regards SARS-CoV-2, if the virus is transferred to eyes, nose or mouth, it can enter the body and infect the subject [12], therefore avoid touching the face has to be a paramount prevention habit. In crucial contexts as health care settings, frequent face-touching is a potential mechanism of acquisition and transmission. A self-inoculation event may occur if a health care worker fails to comply with hand hygiene rules after patient contact or after contact with the patient’s contaminated environment.

Although consequences on hygiene related aspects are the most evident, they are not the only valid reason to discourage people from touching the face. There exist behavioural disorders which are strictly connected to this repetitive movement. Onychophagia [13] (the habit of biting one’s own nails), trichotillomania [14] (the repetitive pulling of one’s own hair) and dermatophagia [15] (the habitual biting of the skin) are just few examples. From the patient’s point of view, episodes of such bad habits are often “automatic” and occur with little apparent control or awareness. This aspect is further supported by a behavioural observation study undertaken in [16] where 26 subjects were observed and videotape recorded to monitor the occurrences of face-touches. Using standardized scoring sheets, the frequency of hand-to-face contacts with mucosal or nonmucosal areas was tallied and analysed. On average, subjects touched their face 23 times per hour. Of all face-touches, 44% involved contact with a mucous membrane, whereas 56% involved non mucosal areas. Of mucous membrane touches observed, 36% involved the mouth, 31% involved the nose, 27% involved the eyes, and 6% were a combination of these regions.

A common behavioural intervention designed to reduce the manifestations of habit-based disorders is known in literature as habit reversal therapy (HRT) [17]. Its techniques can be organized in five phases: *(i)* awareness training, *(ii)* relaxation training, *(iii)* competing response training, *(iv)* motivation procedures, and *(v)* generalization training. In response to the need to improve awareness, several low-tech strategies including wearing heavy bracelets, perfume, gloves, etc. have been used [18]. On this direction, acoustic, visual, and haptic signals can be employed for providing alerts to users. As an example, in [19] a loud tone was used as a deterrent with a 36-years old woman who had been diagnosed with moderate mental retardation and hair pulling. This study demonstrated how an audio alert upon coming in contact can be experienced as aversive and may contribute to a reduction in bad behaviours.

As a matter of fact, audio and/or visual cues may be ineffective or undesired in some circumstances, especially when vision is temporarily impaired or background noise makes auditory feedback difficult to hear or understand. On the contrary, the sense of touch is not only the most robust and distributed of human senses, but it is also proximal, bidirectional, and private [20]. These features make the haptic channel particularly suitable to convey information in everyday environments, where visual and auditory modalities might be busy to effectively accomplish a task (e.g., vision occupied in finding objects), impaired due to personal protective equipment (e.g., worker wearing headphones), or inappropriate (e.g., student attending lecture, spectator during a public show). Vibrotactile anklets [21], dorsal and waist belts [22], bracelets [23], and rings [24] have been deeply studied and extensively exploited as haptic means for providing information to users. Examples range from encoding complex directional cues to human-robot collaboration, from enhancing human-human social activities, to limb guidance and situational awareness.

**FIGURE 1.**
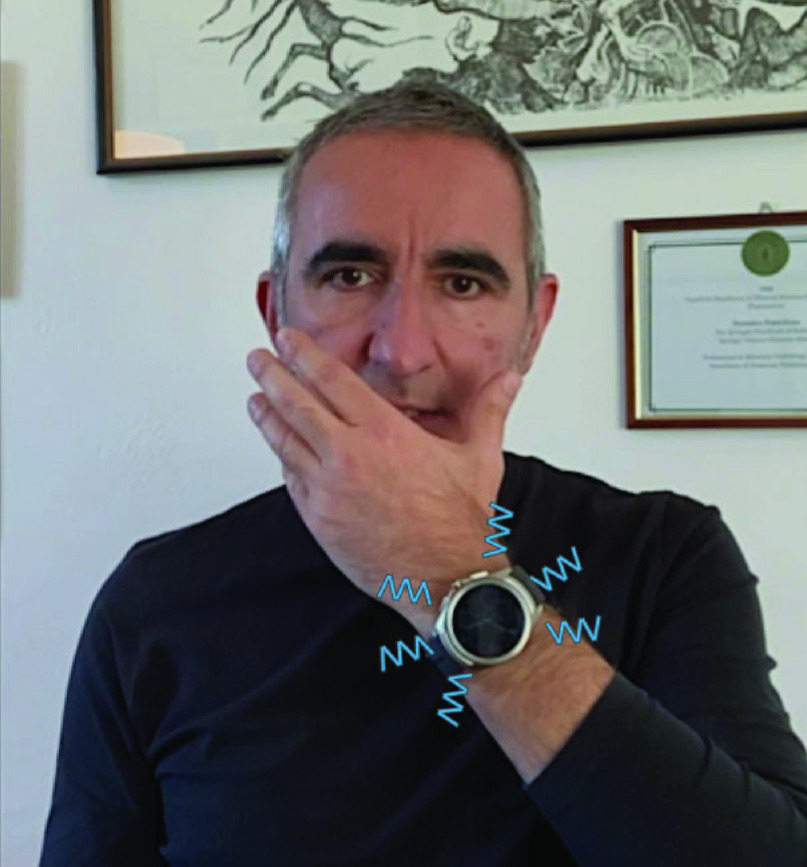
The application No Face-Touch runs on the smartwatch. It estimates hand proximity to face and notifies the user with a vibration whenever a face-touch movement is detected.

In this manuscript, we present “No Face-Touch”, an open project exploiting haptic feedback for suggesting and training people to develop good habits, in order to limit further transmission of SARS-CoV-2, and more in general, to help people become more aware of their face-touching. To systematically detect all the times the hand approaches the face, an automatic system is required. The idea lies on the exploitation of widespread and off-the-shelf devices, such as smartwatches and smart bracelets, to track the human hand and notify the subject in case of face-touching. This solution will minimize the mental effort required to keep hands away from the face, catching also involuntary movements that would take place without the subject noticing. The concept is visually summarized in Figure 1. The choice of the smartwatch as core technology came for a precise reason: we wanted to provide immediate help to people, without the requirement of buying or creating new hardware. From the literature, widely exploited is the use of cameras [25], magnetic technologies [26], and exoskeletons [27] to track arm and wrist pose. Even though recognized as reliable, these setups require expensive and bulky equipment or complex and elaborated installation procedures. On the other hand, several methods have been developed for computing the absolute objects pose (i.e., with respect to the world reference system) by means of Micro Electro-Mechanical Systems (MEMS) sensors, typically embedded in smartwatches [28].

The paper is organized as follows. Section II provides a description of the proposed system from an engineering perspective, including hardware and software specifications. The third section (Section III) presents in detail the algorithms we developed to detect face-touch events. Section IV describes the experiments performed to verify the objectives achievement and reports a-posteriori discussions, enriched with statistical analysis of results. Conclusions are drawn in Section V, along with a brief discussion on the range of possible new development directions that No Face-Touch may enable. Source code repositories, available releases, and compatible devices are listed in Section V. A conclusive Appendix contains the pseudo-code implementation of the algorithms detailed in Section III.

## II. No Face-Touch System

The objective of the No Face-Touch project is identifying whenever the hand gets too close to the face, and alerting the user to stop the current motion. With the aim to develop a ready-to-use and large-scale deployable system, two design guidelines have been followed. Firstly, only technologies already available on widespread devices have been exploited. Secondly, the system implementation is thought to be highly plug-and-play, meaning that no complex installation and/or hardware assembly procedures are needed to let the system work. As a result, No Face-Touch is composed of three elements: *i)* a smartwatch worn by the user; *ii)* an application running on the smartwatch or on the companion smartphone; *iii)* a wearable accessory worn close to the face (like a necklace, a pair of earrings or a pair of glasses) embedding magnets to generate a detectable magnetic field. While the first two elements are essential for the system functioning, a configuration without the third element has been proposed and evaluated as alternative solution.

Two different policies have been developed. In one case, the algorithm takes advantage of data coming from accelerometer and magnetometer sensors, while in the second case only acceleration measurements are used. Indeed, this second method accounts for the fact that many smartwatches do not feature a magnetometer. Although less robust (as reported in Section IV-B), in our regards it was worth proposing an alternative approach to provide support to the widest possible amount of people. From the software point of view, the application has been developed for different platforms to take into consideration the variety of smartwatch brands and operative systems available in commerce.

Obviously, face-touches can occur with motions performed by both left and right arm. Even if the system has been characterized and tested wearing a single device, i.e. monitoring only a single arm, we believe that its validity is not diminished. Indeed, it has been demonstrated that HRT is an efficacious long-term behavioural intervention [29], [30]. Thus, a valid alternative to wear two devices is interchanging the smartwatch position on left and right arm.

## III. Exploited Methods

In this section, we present methods and algorithms implemented for carrying out the different stages of the experimental validation. For each phase, a different software has been developed to collect data from inertial and/or magnetic sensors. While the first evaluation is preparatory for the system functioning, and thus the *ad-hoc* application does not have any use outside the experiment, the algorithms used in the second and third experiments have been released in a public repository as the No Face-Touch application.

In what follows we report the rationale and description of the exploited methods, whereas the experimental evaluations are in Section IV.

### A. Safe and Unsafe Orientations

As a preparatory phase, we identified a range of admissible wrist orientations for face-touch events with the aim of establishing discriminatory conditions for the face-touch detection. Indeed, not all wrist orientations are compatible with natural touches of the face. Anatomical constraints and articular control strategies suggest that, in order to detect the hand approaching the face, we can consider a narrow subset of all the possible wrist orientations [31]. Therefore, starting from theoretical values found in literature, we recruited participants to define the boundaries of the above mentioned subset. The experiment aimed at classifying wrist orientations into two categories:

*safe:* wrist orientations that are not compatible with natural face-touch movement;
*unsafe:* orientations usually assumed by the wrist while the hand approaches the face.

To this end, subjects’ hand movements were measured by means of an *ad-hoc* app running on the smartwatch. The application implements the algorithm described in [32] which, on the basis of the Multiplicative Extended Kalman Filter (MEKF), accurately estimates the body posture with a low-cost wearable setup. In particular, the MEKF estimation proposed in [32] performs a correction step only when the measurements are sufficiently reliable. The resulting system does not suffer from occlusion problems and lightening conditions, and it can be used in indoor and outdoor environments. Moreover, since only accelerometer and gyroscope are used to estimate the orientation, the system can be used in the presence of hard and soft iron and magnetic disturbances, common in smartwatches. The interested reader is referred to [32] for further details.

Boundary values for *safe* and *unsafe* wrist orientations obtained in this experimental phase are adopted in both detection algorithms.

### B. Detection With Magnetometer

**FIGURE 2.**
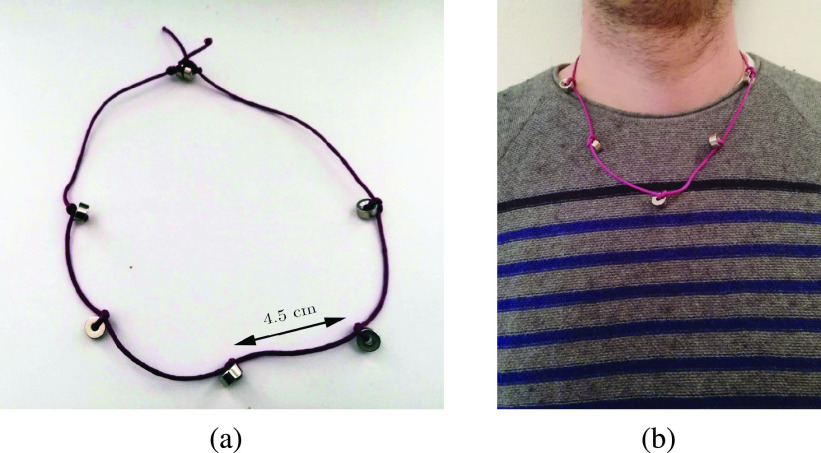
In (a) a handmade magnetic necklace prototype. It contains 5 neodymium magnets, 4.5 cm far from each other. In (b) a user wearing the magnetic necklace.

The proximity between hand and face is estimated thanks to a virtual magnetic barrier generated by the worn magnets (see Section II). We used 5 tiny and low-cost neodymium (N42) magnets (10 mm external diameter, 5 mm thick, 2 kg pull force). We experimentally verified that each magnet can generate a magnetic field of $\mathrm {\sim 420~ \mu \text {T} }$ at 5 cm distance, i.e. a substantial variation with respect to the magnitude of the Earth’s magnetic field which at its surface ranges from 25 to 60 $\mathrm { \mu \text {T} }$ [33]. In a typical scenario the user wears a necklace with 5 magnets far 4.5 cm each other, as shown in Figure 2. Such arrangement is adequate to detect the smartwatch proximity around the necklace, as a result of the significant magnetic field generated by the high performance magnets. Thanks to the calibration procedure, a different number of magnets with different technical specifications can be employed, allowing each user to build his own wearable accessory with great flexibility.

As a matter of fact, devices and objects that interfere with the magnetometer populate almost every daily environment. The Earth magnetic flux is remarkably deflected and modified by ferromagnetic materials, while electronic devices such as computers, mobile phones, and general household appliances generate electromagnetic fields (EMF) that may result in artifacts or relevant fluctuation of the background noise. From this observation arises the need to reinforce the estimation based on magnetometer data with wrist orientation measurements. Results of preliminary experiments (Section IV-A) revealed that exploiting roll and pitch angles and neglecting yaw orientation is enough to predict if the hand is reaching an unsafe position. Moreover, the robustness granted by the MEKF algorithm in Section III-A comes at the cost of heavy computations. Given the target devices and the related battery consumption issues (the app might work as a background process for several hours), we opted for a simplified estimation procedure exploiting the gravity vector components sampled by the triaxial accelerometer.

The algorithm continuously estimates the orientation of the wrist and checks if it is within the boundary limitations previously defined, i.e. if the hand orientation is safe or unsafe. The *safe orientation* condition (cfr. Section III-A) relies on biomechanical constraints defined in the preparatory experiments, thus we can assume that the hand is far from the face whenever this condition is met. In this case, the app retrieves information about the baseline magnetic field around the user, which is an essential step for discriminating variations due to sensor proximity to the magnets when the wrist orientation is unsafe.

Magnetic field measurements occur at 100 Hz and are used to update a 50-elements buffer that stores information about the environment. This method allows to always keep track of the properties (average value and variance) of the magnetic field around the user, so that a robust threshold approach can be applied. On the contrary, if the current wrist orientation is considered *unsafe*, the magnetic field measurements are compared with the current model of the magnetic field around the user. If the deviation from the baseline of the sensed magnetic field exceeds the standard deviation ($std$) multiplied by a constant factor $\alpha $, then the smartwatch is considered too close to the face and a vibratory alert is triggered to stop the current hand movement. The vibration is interrupted after the face-touch conditions are no longer met. As far as it concerns the value of $\alpha $, by considering the distribution of magnetic field around the user as Gaussian, such factor should be 3. In fact, from the theoretical point of view, given mean and standard deviation of a Gaussian distribution, almost 99.7% of values would fall in the $mean\pm 3\!\cdot \! std$ interval. On the other hand, because of the irregularity of the magnetic noise, this value has to be increased to efficaciously detect the presence of the magnets. Therefore, the multiplication factor is defined after a calibration procedure (lasting 5 seconds). During this phase the user is asked to extend and move his arm in front of him for 2 seconds and slowly move it toward the necklace, reaching a distance of about 20 cm far from the latter in the remaining 3 seconds. In the first 2 seconds, the procedure computes the environmental mean and standard deviation, then in the following step the maximum value is recorded. As a result, $\alpha $ is calculated as the fraction between the maximum value stored in the last 3 seconds and the standard deviation computed in the first part of the calibration. The distance adopted in the calibration phase is recommended as an indicative value for ensuring that the collected data do not include relevant variations of the magnetic field caused by the presence of the permanent magnets. Indeed, considering that the magnetic field intensity decreases proportionally to the squared distance from the source, the influence of the magnetic accessory is considered negligible when the sensor is at a distance of 20 cm from the necklace. In practice, the user can estimate this distance by touching the central magnet with the index fingertip.

A pseudo-code implementation of the method is reported in Algorithm 1.

Algorithm 1 **Detection With Magnetometer**
**Initialization:**
$\theta = 0, \phi = 0,\Phi =0$, $\bar {\Phi }=0$, $\hat {\Phi }=0$, $\alpha = 0$, $N= 200$, $M=50$, *buffer* $= \emptyset $, *alert* = *False*
**Calibration:**
Phase 1: $\triangleright $arm far from the magnets
**while** $time\leq $2$~seconds$ **do**
$[m_{x}~m_{y}~m_{z}] \gets $read magnetometer
$\Phi \gets \sqrt {m_{x}^{2}+m_{y}^{2}+m_{z}^{2}}$
**if** $i < N$ **then**
buffer.appendLast($\Phi $)
**else**
*buffer*.removeFirst()
*buffer*.appendLast($\Phi $)
**end if**
$i\gets i+1$
**end while**
$\bar {\Phi } \gets \frac {1}{N} \sum _{i=1}^{N} \mathit {buffer}(i)$
$\sigma _\Phi \gets \sqrt {\frac {1}{N-1} \sum _{i=1}^{N} (\mathit {buffer}(i) - \bar {\Phi })^{2}}$
Phase 2: $\triangleright $move the watch closer to magnets
**while** $time\leq $3$~seconds$ **do**
$[m_{x}~m_{y}~m_{z}] \gets $read magnetometer
$\Phi \gets \sqrt {m_{x}^{2}+m_{y}^{2}+m_{z}^{2}}$
**if** $\Phi > \hat {\Phi }$ **then**
$\hat {\Phi } \gets \Phi $
**end if**
**end while**
$\alpha \gets (|\hat {\Phi }-\bar {\Phi }|)/\sigma _\Phi $
**Monitoring:**
loop
$[a_{x}~a_{y}~a_{z}] \gets $read accelerometer
$\theta \gets \mathop {\mathrm {arctan2}}\nolimits (a_{y}, a_{z}) \cdot 180/\pi $
$\phi \gets \mathop {\mathrm {arctan2}}\nolimits (-a_{x}, \sqrt {a_{y}^{2} + a_{z}^{2}}) \cdot 180/\pi $
$[m_{x}~m_{y}~m_{z}] \gets $read magnetometer
$\Phi \gets \sqrt {m_{x}^{2}+m_{y}^{2}+m_{z}^{2}}$
**if** $\theta _{\textrm {min}}\! < \!\theta < \!\theta _{\textrm {max}}$ and $\phi _{\textrm {min}}\! < \!\phi < \phi _{\textrm {max}}$ **then**
*safeOrientation* $\gets $ False
**else**
*safeOrientation* $\gets $ True
*buffer*.removeFirst()
*buffer*.appendLast($\Phi $)
$\bar {\Phi } \gets \frac {1}{N} \sum _{i=N-M}^{N}$ buffer(i)
$\sigma _\Phi \gets \sqrt {\frac {1}{N-1} \sum _{i=1}^{N} (\mathit {buffer}(i) - \bar {\Phi })^{2}}$
**end if**
**if** ! *safeOrientation* and $(|\Phi - \bar {\Phi }|/\sigma _\Phi) > \alpha $ **then**
**alert** $\gets $ *True*
**else**
*alert* $\gets $ *False*
**end if**
**end loop**

### C. Detection Without Magnetometer

A different version of the face-touches detection algorithm has been developed to be functional also in simpler wearable devices not embedding a magnetic field sensor. Examples of these devices are the common and widespread fitness smart-bands that can be exploited with a companion smartphone. Similarly to [34], the proposed algorithm leverages inertial measurements only. The developed policy aims at recognizing wrist motions that could be associated with a face-touch. The main assumption is that only a subset of all the possible hand motions terminates in a contact with the face. By taking advantage of biomechanical constraints of the human body, we can predict if the current combination of wrist orientation and acceleration profile leads to a touch. A pseudo-code implementation of the algorithm is provided in Algorithm 2, while its functioning is detailed in the next lines.

Algorithm 2 **Detection Without Magnetometer**
**Initialization:**
$\theta = 0$, $\phi = 0$, $\dot {\phi } = 0$, $\beta = 0$, $N = 200$, $M = 50$, $i = 0$, *buffer* = $\emptyset $, *slope* = $\emptyset $, *rising* = *False*, *safeOrientation* = *True*, *alert* = *False*
$\triangleright $ **Calibration:** arm still during calibration
**while** $time\leq $2$~seconds$ **do**
$[a_{x}~a_{y}~a_{z}] \gets $read accelerometer
$\theta \gets \mathop {\mathrm {arctan2}}\nolimits (a_{x}, a_{y}) \cdot 180/\pi $
$\phi \gets \mathop {\mathrm {arctan2}}\nolimits (-a_{x}, \sqrt {a_{x}^{2} + a_{y}^{2}}) \cdot 180/\pi $
$\dot {\phi } \gets $compute $\dot {\phi }$
**if** $i < N$ **then**
*buffer*.appendLast($\dot {\phi }$)
**else**
*buffer*.removeFirst()
*buffer*.appendLast($\dot {\phi }$)
**end if**
$i\gets i+1$
**end while**
$\bar {\dot {\phi }} \gets \frac {1}{N} \sum _{i=1}^{N} \mathit {buffer}(i)$
$\sigma _{\dot {\phi }} \gets \sqrt {\frac {1}{N-1} \sum _{i=1}^{N} (\mathit {buffer}(i) - \bar {\dot {\phi }})^{2}}$
$\beta \gets 3\cdot \sigma _{\dot {\phi }} $
**Monitoring:**
**loop**
$[a_{x}~a_{y}~a_{z}] \gets $read accelerometer
$\theta \gets \mathop {\mathrm {arctan2}}\nolimits (a_{y}, a_{z}) \cdot 180/\pi $
$\phi \gets \mathop {\mathrm {arctan2}}\nolimits (-a_{x}, \sqrt {a_{y}^{2} + a_{z}^{2}}) \cdot 180/\pi $
$\dot {\phi } \gets $compute $\dot {\phi }$
*slope*.deleteFirst()
**if** $\dot {\phi }> \beta $ **then**
*slope*.appendLast(1)
**else**
*slope*.appendLast(−1)
**end if**
$\overline {slope} \gets \frac {1}{N} \sum _{i=N-M}^{N} \mathit {slope}(i)$
**if** $\overline {slope} > 0$ **then**
*rising = True*
**else**
*rising = False*
**end if**
**if** $\theta _{\textrm {min}}\! < \!\theta < \!\theta _{\textrm {max}}$ and $\phi _{\textrm {min}}\! < \!\phi < \phi _{\textrm {max}}$ **then**
*safeOrientation* $\gets $ *False*
**else**
*safeOrientation* $\gets $ *True*
**end if**
**if** ! *safeOrientation* and *rising* **then**
*alert* $\gets $ *True*
**else**
*alert* $\gets $ *False*
**end if**
**end loop**

As in the first part of the previous algorithm, gravity vector components ($a_{x}, a_{y}, a_{z}$) sensed by the smartwatch are used to reconstruct roll and pitch angles of the wrist in world reference frame. In the initialization phase, the user is asked to maintain the hand in resting position for 2 seconds while a calibration procedure acquires inertial data and computes the correspondent wrist orientation. This process provides an estimate of the sensor’s noise, which is necessary to discriminate angle variations due to slow movements from random processes. Thus, a threshold ($\beta $) is obtained from the standard deviation of the variation of the orientation and it is used to distinguish angle variations due to movement from noise or artifacts.

Once the calibration is done and the threshold is defined, the algorithm monitors both the wrist orientation and the variation of the wrist pitch. More in detail, at each iteration, the difference between current pitch value and previous one is calculated and stored in an array named *slope* as follows: if the last variation is greater than $\beta $, then *‘1’* is appended, otherwise *‘-1’*. The average value of the last *50* samples of *slope* reveals whether the hand is moving upwards. If the hand was rising and the wrist orientation is within the unsafe range, then the user receives a vibratory alert. The vibration is interrupted after these conditions are no longer met. Clearly, there are many gestures that correspond to the subset of movements identified by the conditions defined. Hence, in this algorithm the number of false positive is expected to be higher with respect to the algorithm exploiting the magnetometer sensor.

## IV. Experimental Validation

In this section we detail the stepwise validation undertaken to assess No Face-Touch functioning. A list of the adopted devices and their specifications is reported in Table 1. For each step, experimental protocol, setup, and results are described in what follows. The study was approved by the Local Institutional Ethics Committee. Each subject gave her/his written informed consent to participate and was able to discontinue participation at any time during experiments. The experimental evaluation protocols followed the declaration of Helsinki, and there was no risk of harmful effects on subjects’ health. Data were recorded in conformity with the european General Data Protection Regulation 2016/679, stored locally on the smartwatch with anonymized identities (i.e., Subject 1, Subject 2), and used only for the post processing evaluation procedure. Please note that no sensible data were recorded (only date and time of face-touch events detected). A detailed summary of the carried out experimental sessions is reported in Table 2.

**TABLE 1:** List of the Devices Used for the Experimental Evaluation

Smartwatch Model	Magnetometer	Accelerometer	Weight	Operative System	Battery
LG Urbane 2	Yes	Yes	92 g	Wear OS 2.1	Li-Ion 570 mAh
Asus ZenWatch 3	Yes	Yes	48 g	Wear OS 2.1	Li-Ion 341 mAh
Huawei Watch 2	Yes	Yes	57 g	Wear OS 2.1	Li-Ion 420 mAh
Apple Watch Series 4	No	Yes	48 g	WatchOS 6.1	Li-Ion 292 mAh

**TABLE 2:** Summary of the Experiments. The Pattern Set Describes the Number of Trials Performed by Each Participant. The 2 Conditions Stated in E2 Pattern Set are the Magnetometer-Based and Inertial-Based Algorithms, While in E3 the 2 Conditions Differ by the Enabling of Haptic Alerts

Experiment	Pattern set (per participant)	Location	Number of participants
Preparatory (E1)	20 trials	Lab setting	10
Algorithm Comparison (E2)	2 conditions $\times$ 2 set of 30 trials each	Lab setting	10 (6 from E1)
Real Scenario (E3)	2 conditions $\times$ 6 trials lasting 4 hours	Home setting	10 (5 from E2)

### A. Preliminary

As a first step, we investigated wrist orientations leading to face-touches. As anticipated in Section III-A, we consider *safe* an orientation that is not compatible with a face-touch event. Conversely, we indicate as *unsafe* the wrist orientations consistent with a contact between the hand and the face. The experiment was held in laboratory settings. Ten healthy subjects (7 males and 3 females, aged 24-59, 6 right-handed and 4 left-handed) were recruited. None of them reported any known deficiency in perception abilities or physical impairments. Participants were informed about the procedure and trained on the experimental system handling.

The smartwatch adopted was a LG Urbane 2. Both right-handed and left-handed users wore the smartwatch on their non dominant arm, in accordance with [35]. Then, they were tasked to freely touch their face 20 times for at least 5 s with the hand wearing the smartwatch. Hand motions were recorded using a RGB camera (12 Mp, 4608 $\times$ 2592 pixel, F 2.2, 30 fps) and tracked by means of an *ad-hoc* application implementing a customized version of the algorithm presented in [32] and running on the smartwatch.

Results

**FIGURE 3.**
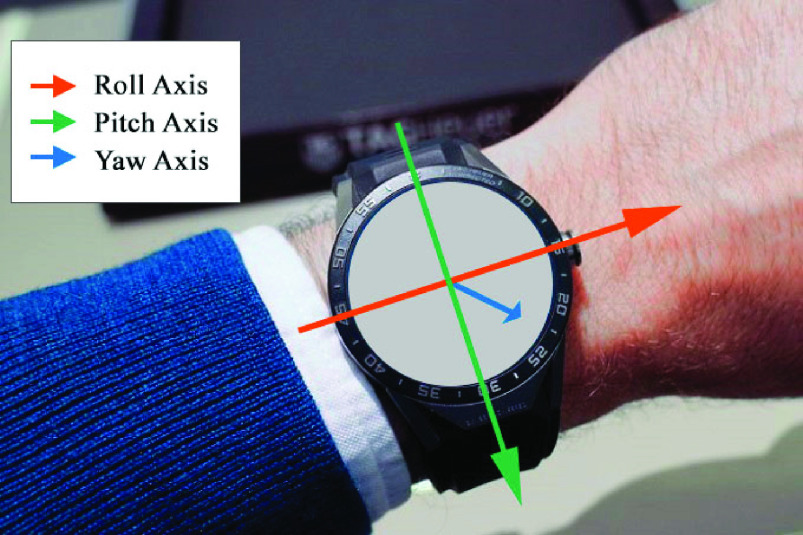
The smartwatch local reference system is defined by sensors axes: the longitudinal axis (roll), transverse axis (pitch), and vertical axis (yaw) are depicted in red, green, and blue, respectively.

In the post-processing phase, video recordings were synchronized with inertial data recorded by the app and used as reference to identify angular patterns measured during the face-touches. Wrist angles were estimated for each timeframe and selected by visual inspection of the video. The set of angles compatible with face-touches were used to classify *safe* and *unsafe* orientations. Considering the Earth as a reference system, we used a common aeronautical inertial frame where the x-axis points north, the y-axis points east, and the z-axis points down, aligned with the gravity. We will call this as North-East-Down (NED) reference frame. The terms used to represent a given orientation are roll ($\theta $), pitch ($\phi $), and yaw ($\psi $) for rotations around x-, y-, and z- axes, respectively. The sensor reference system for the smartwatch employed is depicted in Figure 3. Results of this preparatory phase assessed that any values of yaw is compatible with a face-touch. This is an obvious result, since yaw rotations describe orientation changes around the z-axis (i.e., the Earth gravity vector). For what concerns roll and pitch, inertial data recorded during the experimental trials were analysed to retrieve the set of angles compatible with a face-touch. The following values are considered compatible with unsafe orientations for right-handed users:

\begin{align*} -90^\circ=&\theta _{\textrm {min}} < \theta < \theta _{\textrm {max}} = 70^\circ \\ 30^\circ=&\phi _{\textrm {min}} < \phi < \phi _{\textrm {max}} = 100^\circ\tag{1}\end{align*}

For what concerns left-handed users, obtained values were very close to the ones identified in (1), so for the sake of simplicity, we decided to exploit the same ranges. Due to the opposite orientation in wearing the watch, the values are symmetrical:

\begin{align*} -70^\circ=&\theta _{\textrm {min}} < \theta < \theta _{\textrm {max}} = 90^\circ \\ -100^\circ=&\phi _{\textrm {min}} < \phi < \phi _{\textrm {max}} = -30^\circ\end{align*}

### B. Algorithms Comparison

As a further step towards the goal of evaluating the system, we compared the performance of the two proposed algorithms. An experimental validation was conducted in laboratory settings to measure the accuracy in detecting potential face-touches.

Ten subjects (7 males and 3 females, aged 21-61, all right-handed) were involved in this experiment. Three different smartwatches running the app in background were used: a LG Urbane 2, an Apple Watch series 4, and an Asus ZenWatch 3. Participants were tasked to perform two trials: *i)* attempt to touch their face 30 times, and *ii)* simulate 30 common gestures of Activity of Daily Living (ADL). The set of ADL was previously selected from the list proposed in [36], according to the criteria of choosing gestures similar to a face-touch (e.g., eating with a spoon, drinking from a mug, hair-combing, putting on a t-shirt). Subjects were asked to wear the smartwatch on their non dominant arm and perform twice both trials (face-touch and ADLs gestures, respectively), once with the magnetometer-based algorithm and once with the accelerometer-based algorithm. The order of gestures and conditions was pseudorandomly selected at the beginning of each experiment. Participants were recorded using an RGB camera and data were post-processed to evaluate and compare the accuracy of the algorithms. *Correct detections* and *false positives* were used as metrics. To estimate the number of correctly detected face-touches, we measured the number of alerts generated by the system and we compared them with the total number of face-touch gestures performed by the user. Similarly, the number of notifications displayed by the device while performing other motions is reported as false positive i.e., number of undesired vibratory alerts.

Results

In Table 3 we reported the average percentages of correct detections and false positives computed among the users. Results confirmed the hypothesis, i.e. the algorithm relying on both accelerometer and magnetometer sensors is more robust and more accurate than the one exploiting only accelerometer measurements. It is worth pointing out that the higher percentage of correctly detected scored by Algorithm 2 is biased by the large amount of false positives. In fact, this algorithm is prone to exceed in alerting the user, regardless the motion.

**TABLE 3:** Algorithms Accuracy Comparison. Correctly Detected Expresses the Percentage of Alerts Provided Over the Total Face-Touch Gestures, Whereas False Positives Indicates the Ratio of Generated Vibrations With Respect to the Total Number of Activity of Daily Living Executions

	Correctly Detected	False Positives
Algorithm 1	91.3%	3.2%
Algorithm 2	92.6%	38.1%

On the basis of the obtained results, we decided to continue the experimental validation using only the algorithm comprising the magnetometer sensor.

### C. Real Scenario

Once the algorithm accuracy had been assessed, we performed an experimental campaign to evaluate the effectiveness of the app. We formulated two hypotheses:

i) the system reduces the duration of face-touches (immediate effect due to notification of gesture detection);
ii) the system reduces the amount of face-touches (medium-to-long term effect).

In order to test No Face-Touch for both hypotheses, experiments were carried out using two conditions: detection notified with a vibration (${V}$) and detection not notified (${N}$). The latter has been used as a control condition for the statistical analysis.

Ten subjects (6 males and 4 females, aged 23-65, all right-handed) were involved in this phase. Three different smartwatches running the application in background were used: a LG Urbane 2, a Huawei Watch 2, and an Asus ZenWatch 3. All the considered devices embed magnetometer sensors with comparable accuracy. Participants were asked to wear the smartwatch on their non dominant arm for 6 days, 8 hours per day: from 9 to 13 (A.M. time interval) and from 15 to 19 (P.M. time interval). Users performed the same actions (working or activities of daily living) in all the considered days. The experiment was carried out mainly in home settings, although participants were free to go outside for shopping or working purposes. All the events in which the users crossed the virtual magnetic barrier and the related duration were recorded in a textual log file.

The two monitoring modalities (with and without haptic feedback) were used once per day by each user, following a pseudo-randomly generated sequence. During the post processing phase, potential face-touch events recorded by the app were classified depending on their duration. Indeed, since we could not measure the hand position, we exploited a time threshold to distinguish a Touch Attempt (TA) from an Happened Contact (HC). We experimentally assessed that face-touch attempts last less than a second, then we considered as TA all the recorded events in line with aforementioned short-lasting time. On the contrary, whenever the hand remained in the alerting state for more than a second, the event was classified as an HC, and the exceeding time was recorded as the HC duration. This classification was useful to analyse the effects of the alert (vibration) on subjects’ behaviour. In addition, since it is of interest of this work preventing unsafe behaviour and training people to develop good habits, we considered worthy of analysis both number of TAs and number of HCs.

After the conclusion of the experiment, an online anonymous survey was requested to each subject. The survey collected opinions and feedback with a single open-end question proposed within a text box, in which respondents could formulate their own answers in less than 100 words. To highlight the system efficacy in preventing and reducing face-touches, a comparison among the two modalities was carried out by means of a statistical analysis of the data. Multiple paired-samples t-tests were conducted to determine whether there are statistically significant differences between the metrics of interest over the two feedback modalities (${V}$ and ${N}$). Then, two paired-samples t-tests were performed to determine whether there was a statistically significant effect of the condition on the number of HC/hour and HC duration in the two modalities.

Results

In what follows, data are mean ± standard deviation, unless otherwise stated.

**FIGURE 4.**
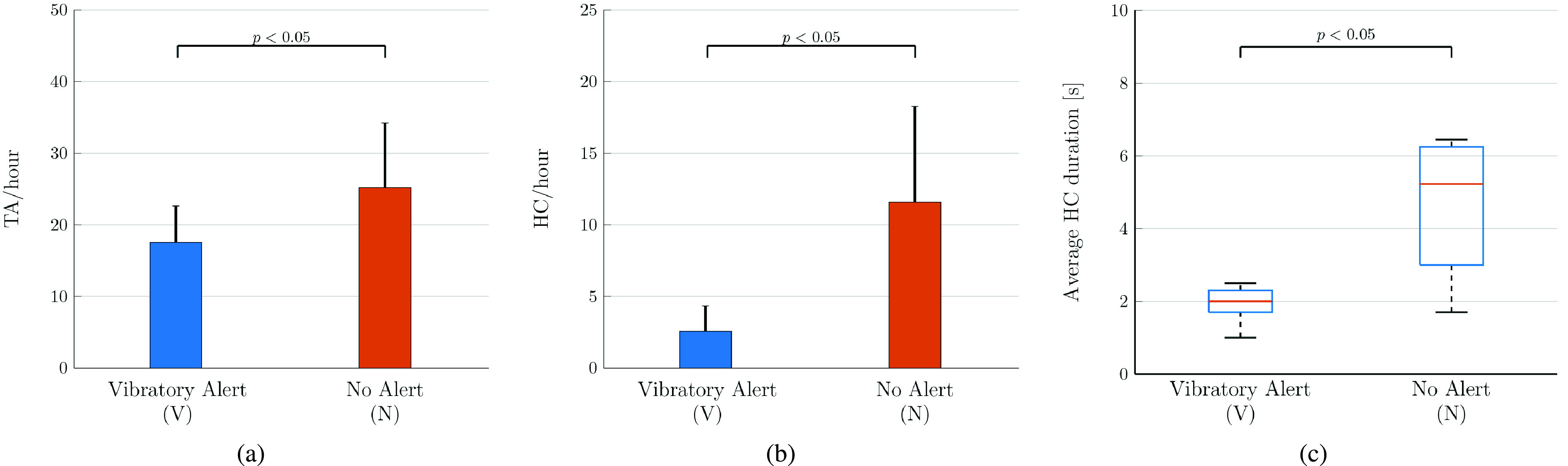
Experimental validation results. Mean and standard deviation of the two modalities outcomes are plotted. The p-values, computed with paired-samples t-test, are reported above the bar charts. Number of touch attempts (TA) per hour are reported in (a), while in (b) each bar represents the average amount of happened contacts (HC) per hour. Finally, (c) represents the distributions of HC duration for the two conditions.

Firstly, a-posteriori analysis with paired samples t-test determined that there is a statistically significant mean reduction in number of TA per hour. The assumption of normality was not violated, as assessed by Shapiro-Wilk’s test ($p = 0.682$). Unsafe gestures were observed more often when no vibration was provided (25.18 ± 9.03 TA/hour) than in case of vibrotactile notification (17.53 ± 5.10 TA/hour). We verified a statistically significant reduction of 7.65 TA/hour, $t(9) = 2.55$, $p= 0.031 < 0.05$. In Figure 4, mean and standard deviation comparison is visually reported.

For what concerns the HC/hour ratio, no outliers were detected and the assumption of normality was not violated, as assessed by Shapiro-Wilk’s test ($p = 0.303$). Number of face-touches was smaller when subjects used the system with the haptic feedback (2.56 ± 1.76 HC/hour) compared to the system with no notification (11.59 ± 6.68 HC/hour). A statistically significant increase of 9.02 HC/hour was confirmed by the test, $t(9) = 4.77$, $p = 0.001 < 0.05$. Such results are also depicted in Figure 4b.

Regarding the analysis of touch duration, data did not pass ShapiroWilk normality test in both condition, as visible in Figure 4c. After squareroot transformation, the normality condition was satisfied (ShapiroWilk normality test $p > 0.05$). The paired-samples t-test assessed a statistically significant difference between duration of touches using or not the vibration alerting. The duration of face-touches was significantly reduced, from a median value of 5.21 seconds when participants were not notified to 2.03 seconds if the smartwatch vibration was enabled, $t(9) = 2.795$, $p = 0.021 < 0.05$.

As additional analysis, we estimated the efficacy of the system in preventing happened contacts considering the number of gestures that ended with a face-touch (both with and without vibratory notification. Results demonstrated that the 86.3% of unsafe gestures were interrupted in time when the vibrating alert was active. This means that after the notification, subjects stopped their gesture in less than 1 s. Longer actions instead were classified as HC.

Finally, a *post-hoc* test made by means of the software G*Power assessed that to have data from 10 participants was enough for a statistical power of at least 0.8 for all the conducted tests.

Qualitative results were derived by analysing final partecipants’ feedbacks.^1^ All the users were enthusiast of the system. Most of them pointed out that, when notified, the gesture was continued only in case the face-touch was strictly necessary, e.g. in case of itches. Additionally, users reported that in such cases the gesture was conducted paying greater attention. Moreover, participants particularly appreciated the fact that they could use the system without wearing bulky hardware and that the equipment could be easily hidden. The fact that the necklace is a passive device and can be worn under the shirt was highlighted three times. A negative flaw of the system, on which the majority of the users agreed, was the reduction of the smartwatch battery lifetime. Indeed, running the application in background rapidly discharges the device, that needs to be recharged once per day.^1^Answers are available at http://sirslab.dii.unisi.it/nofacetouch/answers.html

## V. Conclusion and Future Work

With this work we propose a ready-to-use solution to discourage people from touching their face in dangerous or hazardous environments, whose short/mid-term effectiveness has been proven by the experiment results. The proposed system is not only a precious aid to prevent further infection of COVID-19, but it can also help to improve face-touch awareness in patients undergoing habit reversal therapy. On the basis of the literature review presented in the introduction, we can hypothesise that a prolonged exploitation of No Face-Touch will lead people in reducing face-touch occurrences also in the long term. We will examine in depth this aspect in a future work.

The presented results pave the way for numerous interesting research directions. As an example, the same approach can be exploited also to limit nail biting behavioural disorder. In such specific case the wrist orientation range can be further reduced to have a more precise hand position estimation.

One of the great advantages of the proposed framework is that it does not require complex hardware equipment or software implementation. With few efforts in adapting, No Face-Touch can be integrated in a wide group of smart-bands embedding magnetometer sensors, or alternatively, a simple DIY bracelet can be built with off-the-shelf electronic components. Such cheap alternatives enable the user to wear a pair of devices, one per arm, duplicating the effectiveness.

## Appendix Algorithms

In this section we report the pseudo-code implementation of the algorithms detailed in Section III-B and Section III-C.
